# A tale of two tracts: Comparison of the survival rates of upper and lower GIT cancers by age, gender, and handgrip strength in geriatric population from 12 European countries

**DOI:** 10.21203/rs.3.rs-7839736/v1

**Published:** 2025-11-19

**Authors:** Salman Yousuf Guraya, Rizwan Qaisar, M. Azhar Hussain

**Affiliations:** University of Sharjah; University of Sharjah; University of Sharjah

**Keywords:** Gastrointestinal cancer, handgrip strength, physical capacity, SHARE, survival

## Abstract

**Background:**

Gastrointestinal tract (GIT) cancers are among the most prevalent malignancies worldwide. Due to variations in anatomical locations and biological behaviors of upper and lower GIT cancers, differences exist in their survival and physical capacity domains. Limited large-scale comparative datasets exist for upper and lower GIT cancers, particularly in the geriatric population. This study aimed to compare survival rates and handgrip strength (HGS) between upper and lower GIT cancer European geriatric patients, with specific attention to age and gender differences.

**Methods:**

This longitudinal cohort study used data about the European geriatric population from the Survey of Health, Ageing, and Retirement in Europe (SHARE) across waves 4 to 9. HGS was measured using a dynamometer. Regression analysis was performed using the survival methodology with a Weibull distribution adjusted for age, gender, and HGS.

**Results:**

A total of 33,379 adults aged ≥ 50 were included, comprising 106 participants with upper GIT cancer and 99 with lower GIT cancer. Overall, nine-year survival was 39.6% for upper GIT and 34.3% for lower GIT cancer patients. Lower GIT cancer was associated with significantly higher mortality risk (β = 0.579, p < 0.001). Female gender conferred a protective effect (β = −0.215, p < 0.001), with women showing notably better survival than men in the lower GIT group. Advanced age (≥ 80 years) significantly increased mortality risk (β = 1.105, p < 0.001). Higher HGS was associated with longer survival (β = −0.365, p < 0.001), particularly in upper GIT cancers.

**Conclusion:**

In this study, patients with lower GIT cancers had worse survival compared to upper GIT cancers, particularly among older men with low HGS. Women with lower GIT cancers had a survival advantage over men, while HGS had a stronger positive predictive value in upper GIT cancers. These distinctions call for personalized and targeted assessment and management plans for GIT cancers.

## Background

Gastrointestinal tract (GIT) cancers have a high incidence rate and mortality, accounting for 5.1 million new cases and 3.6 million new deaths in 2020 worldwide [1]. These cancers may primarily be classified as cancers of the upper (oral cavity, pharynx, esophagus, stomach, liver, and pancreas) and lower (colorectal) cancers. Both cancers have unique epidemiology, risk factors, and clinical outcomes. Such variation is evident by different 5-year survival rates; 65% for colorectal cancers and 33%, 21%, and 12% for gastric, esophageal, and pancreatic cancers, respectively [2]. According to the available evidence in medicine, the treatment efficacy of GIT cancers is primarily determined by survival rates and cancer-related signs and symptoms, while overlooking the physical capacity of the patients. The physical capacity of patients with GIT cancers is a significant determinant of their prognosis and survival and, to some extent, signifies the outcomes of management plans [3].

Most GIT cancers, their surgical therapies, and chemotherapies cause significant myotoxicity with adverse effects on the physical independence of the patients [4]. Specifically, these patients face difficulty in performing physical activities, such as toileting, walking, dressing, and rising from a chair. The impairment of such activities is established as an independent risk factor for recurrence and mortality of patients with lower GIT cancers [5]. Therefore, it may be imperative to monitor the physical independence and activities of daily living in GIT cancer patients as markers of disease progression. However, the relevant data is often self-reported and have considerable subjective variability.

The handgrip strength (HGS) objectively measures the quality of skeletal muscle in cancer patients [6]. HGS is adversely affected by chemotherapy in GIT cancers and can be a reliable tool to predict postoperative complications [7] and survival [8] in GIT cancer. Thus, for GIT cancers, a combination of HGS with the activities of daily living may provide a comprehensive assessment of physical capacity of the affected patients. In GIT cancers, it may be critical to integrate physical capacity with the clinical efficacy of various therapies to obtain a broader perspective on the disease progression. However, several covariates, such as assessment settings (domestic versus clinical), demography and age of patients, racial and ethnic profiles, and socioeconomic status, may each influence the physical capacity of such cohort of patients. However, most relevant studies are performed in the context of regional settings and may not provide a representative dataset. The evaluation of a large dataset of patients with statistical adjustment for covariates may provide more relevant results for physical capacity.

In our study, we used the dataset from the standardized Survey of Health, Ageing, and Retirement in Europe (SHARE), which contains a repository of the longitudinally conducted panel of geriatric adults aged 50 or above across multiple European countries [9]. We compared and analysed age and gender specific data of patients with upper and lower GIT cancers for difficulties performing various routine physical activities, HGS, and survival rates after diagnosis. We hypothesized that the patients with upper GIT cancers exhibit more significant difficulties in performing activities of daily living, and reduced HGS than those with upper GIT cancers.

## Materials and methods

The study includes the geriatric population from the SHARE survey, a representative multi-disciplinary panel data study of individuals aged at least 50 years [9]. SHARE is an international collaborative effort involving nearly all European countries that collects comprehensive data through in-person interviews. The individual-level information covers important dimensions of respondents’ life-stories, including demographic characteristics, socioeconomic conditions, living arrangements, and aspects of the public and personal health situation. The baseline data for this study were obtained from the fifth wave of SHARE conducted in 2013. Still, information from wave 4 (interviews during 2010–2012) was also included: only people without cancer in wave 4 and diagnosed with cancer in wave 5 were included. Subsequent waves, namely wave 6 (2015), wave 7 (2017), wave 8 (2019/2020), and wave 9 (2021/2022), served as follow-up surveys, allowing for the examination of changes over time.

SHARE study wave 5 collected information on cancer in the questionnaire, after showing the respondent a list of 20 diseases/2 other options, where cancer was identified through the question: “Has a doctor ever told you that you had/Do you currently have any of the conditions on this card? With this we mean that a doctor has told you that you have this condition, and that you are either currently being treated for or bothered by this condition Please tell me the number or numbers of the conditions”. Among the possible answers were: “Cancer or malignant tumor, including leukemia or lymphoma, but excluding minor skin cancers”. If cancer was chosen the respondent was asked: “In which organ or part of the body have you or have you had cancer?”. Possible answers included 22 specific organs and the category “other organ”. Upper GIT cancer is present when the respondent chooses the following six organs: Oral cavity, other pharynx, esophagus, stomach, liver, or pancreas. Lower GIT cancer is present when the respondent said the organ was: the colon or the rectum. Subjects were followed in subsequent waves 6, 7, 8, and 9, and it was identified when the person was no longer in the survey due to death. It was thus possible to estimate for how long subjects survived after being diagnosed with a specific type of the two mentioned cancers for those who died during waves 6–9. The rest are right-censored, i.e., they were still alive in wave 9 but will eventually pass away. This censoring means that traditional regression methodologies cannot be applied, and we estimate the survival of patients using duration analysis.

HGS was measured using a hand-held dynamometer (Smedley, S Dynamometer, TTM, Tokyo, 100 kg) [10]. Patients with swelling or inflammation, severe pain or recent injury, or a recent surgery to the hand were excluded from the study. Participants were instructed to press the dynamometer with their left and right hands, performing two repetitions with each hand. If a participant could not use with one hand, measurements were only taken from the other hand. The test instructions required the participants to maintain an upright posture with their upper arm parallel to their torso and their lower arm perpendicular to their torso. If necessary, the test could also be performed in a sitting position. The highest recorded value from these four measurements was used in the subsequent analysis. Low HGS was defined based on gender-specific thresholds, following the guidelines by the European Working Group on Sarcopenia in Older People (EWGSOP2), with thresholds of 27 KG for males and 16 KG for females [11].

## Statistical analysis

Multiple regression analyses were applied to identify individual characteristics affecting survival after a specific cancer diagnosis. Time t (years) to death was statistically modelled as

$$\text{ln}t=\beta_{0}+\beta_{1}\text{Female}+{\text{AgeGroups}\beta }_{2}\text{HGSGroups}\beta_{3}+z$$

where: Female = 1 if the respondent was a female (and 0 for base category males); age group dummies are 60–69 years, 70–79 years, and 80 + years (50–59 years is the base category); HGS groups are low HGS and missing HGS (high HGS is the base category). Coefficients (vectors) β1, β2, and β3 represent effect on survival for the three individual characteristics. The *z* term is the error following the f(.) distribution with an extreme-value density yielding the Weibull regression model (and exponential model). The hazard (empirically, probability of death at time *t*, given survival till time *t*) *h* and survival (empirically, the probability of survival at time *t*) *S* functions are

$$h\left(t\right)=p•\lambda •t^{p-1}\;\text{and}\;S\left(t\right)=e^{-\lambda •t^{p}},\lambda =e^{-p•\backslash \text{varvec}x\backslash \text{varvec}\beta }$$

where the shape parameter *p* is estimated from the data (σ = 1/*p*). All statistical analyses were performed with the software package STATA 18.0 SE Standard Edition (Release 18. College Station, TX: StataCorp LLC) using the Stata commands stset, sts graph, streg, margins, and marginsplot.

## Results

### Demographic characteristics of participants

Table 1 presents summary statistics for individuals categorized into three groups: those with no cancer, those with upper GIT cancer, and those with lower GIT cancer. This table includes data on gender, age, handgrip strength from wave 5, and survival at the last recorded waves 6–9. Regarding gender distribution, among individuals without cancer, 43.0% were men and 57.0% were women. In contrast, among those with upper GIT cancer, 53.8% were men and 46.2% were women, while for lower GIT cancer, there were 59.6% men and 40.4% women. Regarding age distribution, most individuals were in the 60–69 age group across all cancer categories.

The HGS data analysis showed that most individuals had high strength, but a higher percentage of GIT cancer patients had low strength than those without cancer. Some HGS data were missing, particularly in the lower GIT cancer group. Survival status showed that a more significant proportion of GIT cancer patients passed away compared to those without cancer. The percentage of deceased individuals was highest in the lower GIT cancer group (65.7%), followed by the upper GIT cancer group (60.4%).

### Kaplan-Meier survival results

**Figure 1:**
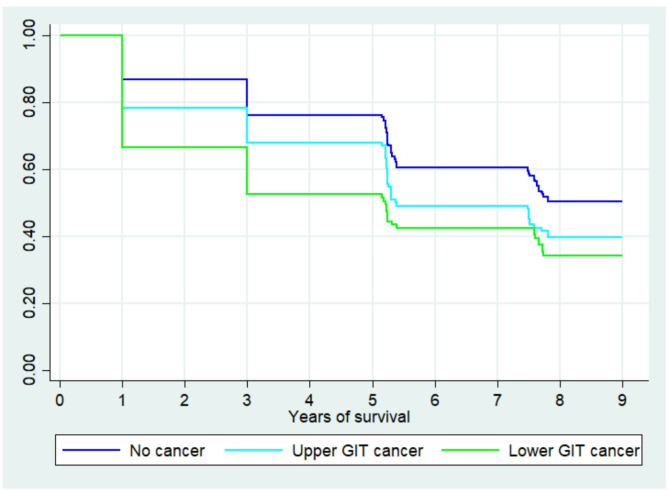
The Kaplan-Meier survival estimates by cancer type.

In Figure 1, the Kaplan-Meier survival curve illustrates the survival probabilities over nine years for individuals with no cancer, lower GIT, and upper GIT cancer. The y-axis represents survival probability from 1.0 (100%) to 0.0 (0%), while the x-axis shows years of survival. All groups start at a survival probability 1.0, meaning 100% of individuals were alive initially. However, survival probabilities declined over time, with apparent differences between groups. By year 3, about 80% of individuals without cancer remained alive, while survival for lower GIT cancer was approximately 70%, and upper GIT cancer survival dropped below 60%. By year 6, survival for upper GIT cancer dropped to almost 40%, while lower GIT cancer remained slightly higher at about 50%. Lastly, by year 9, survival for the no-cancer group remained above 50%, while the lower and upper GIT cancer groups dropped below 40%.

### Regression of survival time

Table 2 outlines the results of survival regression analysis using the Weibull distribution, focusing on how cancer type, gender, age, and HGS affected survival outcomes across various subgroups. The coefficients represent the relationship between each factor and the risk of the event, with significance levels indicated by asterisks. The analysis showed that upper GIT cancer was associated with an increased risk in the overall population. In column one, the coefficient for upper GIT cancer is 0.334**, which indicates a statistically significantly higher risk of death for individuals with upper GIT cancer. In column one for lower GIT cancer, the coefficient is 0.578***, statistically significant at the 1% level. Regarding gender, women were generally associated with a lower risk of death. In column one, the coefficient for women was −0.218***, indicating a significant protective effect compared to men. This negative association was more pronounced in other subgroups, such as column 6 (HGS included continuously), where the coefficient was −2.039***, showing a substantial reduction in risk for women.

For individuals aged 60–69, the reported coefficient was −0.0596**, suggesting a slight reduction in mortality risk, while those aged 70–79 had a positive coefficient of 0.233***, indicating a higher risk. The coefficient for individuals aged 80 and above was 0.895***, emphasizing an increased risk for older individuals. HGS was associated with a reduced risk. In the All group category, participants with high HGS had a coefficient of −0.416***, suggesting that better physical strength is protective for better survival.

### Expected survival time

**Figure 2:**
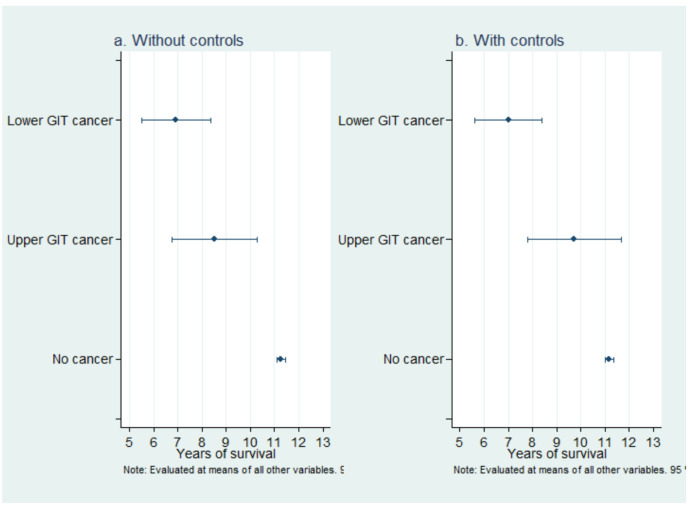
Survival duration by cancer type (a) without and with (b) controls.

Based on the regression analysis results, we can estimate the expected survival of individuals with different demographics and clinical conditions. Figure 2 illustrates the significance of including controls when estimating survival rates in the case of lower GIT cancer. The expected number of survived years differs by more than a year among patients with upper and lower GIT cancers and those without cancers.

**Figure 3:**
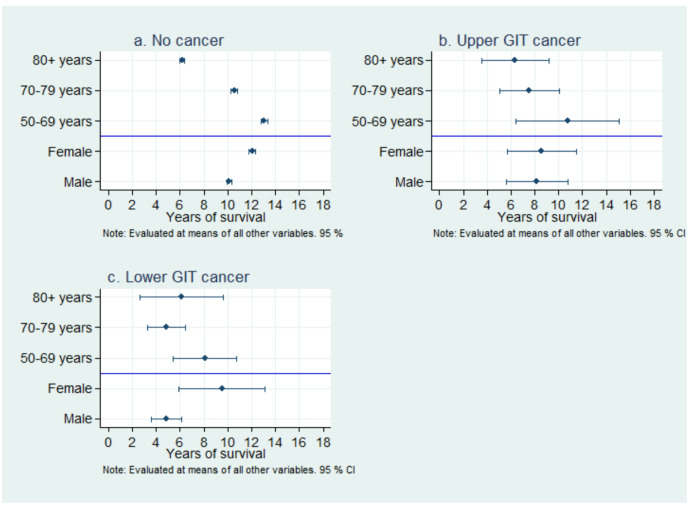
Life expectancy after different cancer diagnoses by gender and age.

Figure 3 presents life expectancy after diagnosing different cancers as classified by gender and age in our study. Individuals without cancers had the highest survival, especially among those aged 50–69 years, while older age groups showed a decline in survival rates. Women tend to live slightly longer than men. Upper GIT cancer significantly reduces life expectancy, with younger patients (50–69 years) having better survival than older individuals. Lower GIT cancer showed slightly better survival than upper GIT cancer, though still lower than those without cancer. Age was found to be a critical factor, as survival declined remarkably in individuals over 80 years.

There were gender differences with overlapping confidence intervals, but not substantial. Figure 3 highlights the impact of cancer type on survival. Not surprisingly, individuals without cancers maintained the longest life expectancy across all age groups.

**Figure 4:**
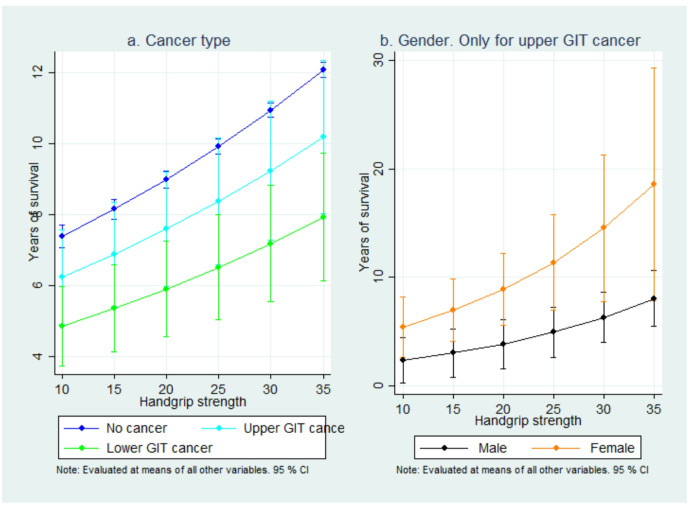
Life expectancy by handgrip strength as analysed separately for cancer types and gender.

Figure 4 illustrates the relationship between HGS and life expectancy, analyzed separately by cancer type and gender. The horizontal axis represents HGS, while the vertical axis shows expected years of survival. In panel a, which focuses on cancer type, individuals without cancer had the highest life expectancy across all levels of HGS. Those with upper GIT cancer had lower survival than the no-cancer group, but higher than participants with lower GIT cancer. Across all groups, stronger HGS was associated with more prolonged survival, highlighting the importance of muscle strength as a predictor of longevity. Panel b focuses on gender differences in survival for upper GIT cancer patients. Women consistently had higher survival rates than men at all HGS levels. The survival gap widened as HGS increases, suggesting that muscle strength is more significant in extending life expectancy for women than men.

## Discussion

In our study, the large representative dataset from SHARE highlights several significant findings about survival in patients with upper and lower GIT cancers. We found that the participants diagnosed with lower GIT cancers experienced lower survival than those with upper GIT cancers. Furthermore, our study reported comparable years of survival for both genders following the diagnosis of upper GIT cancer. Women demonstrated significantly longer survival than men following the diagnosis of lower GIT cancer. The HGS at the time of diagnosis of GIT cancers was found to be a critical influencer, with lower HGS predicting shorter survival. Finally, consistent with previous research, increasing age at the time of diagnosis was significantly associated with reduced years of survival.

The reduced survival of patients with lower GIT cancers compared to those with upper GIT cancers is consistent with previous reports [12] [13]. Thus, the general trend suggests that the anatomical location of the primary tumor significantly influences prognosis. Several factors could contribute to this disparity. Lower GIT cancers often present itself with different patterns of metastasis and may involve distinct molecular subtypes that exhibit varying degrees of aggressiveness [14]. Moreover, the proximity of lower GIT tumors to critical structures and the potential for complex surgical interventions could also play a role in survival outcomes [15]. However, it is important to acknowledge that the SHARE data does not provide details about the extent of cancer spread and the quality of therapeutic interventions.

The observed gender differences in survival rates following the diagnosis of lower GIT cancer, with women exhibiting a more favorable outcome, is a critical observation in our study. While survival in upper GIT cancers appears to be more similar across genders, the divergence in lower GIT cancers suggests a potential interplay of biological and social factors. Biological factors, such as, variations in tumor biology, have been proposed as potential contributors [16]. Additionally, social factors, including differences in healthcare-seeking behavior, treatment adherence, and social support networks, may also play a role. Social drivers of health influence each stage of the cancer care continuum, and racial and ethnic minority patients experience higher rates of adverse social effects such as housing insecurity, few educational opportunities, and low socioeconomic status [17]. The SHARE dataset, while valuable, may not fully capture the complexity of these interactions.

Another key finding of our study was that low HGS at the time of GIT cancer diagnosis predicted shorter survival irrespective of the location of the GIT cancer. HGS is a well-established marker of overall muscle strength and functional capacity, and it has been increasingly recognized as a prognostic indicator in various health conditions, including cancer [18] [19]. Sarcopenia, the loss of muscle mass and strength, is common in older adults, particularly among those with chronic malignancies [20]. Reduced HGS is a key indicator of sarcopenia and can reflect a decline in physiological reserve, making individuals more vulnerable to the adverse effects of cancer and its treatment. Our findings are consistent with studies that have shown the presence of other chronic age-related diseases a predictors for poor physical capacity of the affected patients [21] [18]. Several mechanisms can explain the prognostic values of HGS in our study cohort. A low HGS is frequently associated with sarcopenia and frailty in old age [22]. Sarcopenia can likely lead to increased frailty, reduced tolerance to chemotherapy and surgery, and a higher risk of complications. Furthermore, it may be associated with increased inflammation and metabolic dysfunction, which can promote tumor growth and progression.

Therefore, our study and the available corpus of literature proposes that the assessment of HGS in older adults diagnosed with GIT cancer can be used as a valuable prognostic indicator that can help clinical decision-making. Additionally, interventions to improve muscle strength and physical function such as exercise programs and nutritional support, may improve survival [23]. Further, it is essential to consider that GIT cancers can affect nutritional status and metabolic processes differently. Upper GIT cancers may lead to earlier and more severe malnutrition due to difficulties with swallowing and digestion. Conversely, lower GIT cancers may negatively affect nutrient absorption and bowel function. These functional differences caused by structural variations among GIT cancers lead to sarcopenia and low HGS, influencing overall cancer survival. Therefore, it is proposed that exercise therapies and nutritional support in cancer patients may adequately address sarcopenia, improve the response to cancer therapies, and improve the overall survival after diagnosis [24].

Finally, the finding of our study that increasing age at the time of GIT cancer diagnosis is associated with reduced years of survival corroborates a substantial body of evidence [25]. Age is a well-established risk factor for cancer development, and older individuals often present with more advanced disease and a greater burden of comorbidities. Older patients may also be less tolerant of aggressive cancer treatments, and their overall physiological reserve may be limited. These factors contribute to poorer outcomes in older cancer patients. Our results highlight the importance of considering age as a critical prognostic factor in managing elderly individuals with GIT cancer [26]. Therefore, it is crucial to tailor treatment strategies to the specific needs of elderly patients, considering their overall health status, functional capacity, and treatment preferences. Comprehensive geriatric assessments can help identify older patients at higher risk of adverse outcomes and guide the development of individualized treatment plans.

## Study limitations

This study has several limitations. First, the data were derived from self-reported survey responses, which may introduce recall bias or reporting inaccuracies. Second, the SHARE dataset lacks detailed clinical information such as cancer staging, histological subtypes, treatment modalities, and comorbidities, which could affect survival outcomes. Lastly, despite adjustments for key covariates, residual confounding but unmeasured factors such as nutritional status or functional capacity beyond HGS cannot be overlooked.

## Conclusion

Our study, using data from SHARE, provides valuable insights into the factors influencing survival following GIT cancer diagnosis in the geriatric European population. The findings highlight the significant differences in survival between upper and lower GIT cancers, the gender-specific survival advantage in women with lower GIT cancers, the prognostic importance of HGS, and the adverse impact of increasing age at diagnosis. These results warrant the need for a comprehensive and personalized approach to the management of patients with GIT cancers, with a focus on addressing individual risk factors and tailoring treatment strategies to optimize outcomes.

## Figures and Tables

**Table 1.** Summary statistics of the study population.

		No cancer		Upper GIT cancer		Lower GIT cancer		All	
		n	%	n	%	n	%	n	%
Gender	Male	14,338	43.2	57	53.8	59	59.6	14,454	43.3
	Female	18,836	56.8	49	46.2	40	40.4	18,925	56.7
Age	50–59	10,436	31.5	14	13.2	23	23.2	10,473	31.4
	60–69	11,512	34.7	36	34.0	34	34.3	11,582	34.7
	70–79	7,998	24.1	39	36.8	31	31.3	8,068	24.2
	80+	3,228	9.7	17	16.0	11	11.1	3,256	9.8
Handgrip strength	Low	2,117	6.4	13	12.3	5	5.1	2,135	6.4
	High	28,773	86.7	85	80.2	85	85.9	28,943	86.7
	Missing	2,284	6.9	8	7.5	9	9.1	2,301	6.9
Survival at last wave	Passed away	16,438	49.6	64	60.4	65	65.7	16,567	49.6
	Alive	16,736	50.4	42	39.6	34	34.3	16,812	50.4
Total		33,174	100	106	100	99	100	33,379	100

Source: Own calculations based on SHARE-ERIC datasets from waves 4–9.

**Table 2.** Survival regression analysis for groups with upper and lower GIT cancers and no cancers applying the Weibull distribution.

		All	All	No cancer	Upper GIT cancer	Lower GIT cancer	All	Lower GIT cancer
		(1)	(2)	(3)	(4)	(5)	(6)	(7)
Cancer type	Upper GIT cancer	0.335**	0.133				0.0976	
	Lower GIT cancer	0.579***	0.594***				0.644***	
Gender	Female		−0.215***	_-_0.214***	−0.0608	−0.480	−0.544***	−0.979*
Age	60–69		−0.00671				−0.0745***	0.858*
	70–79		0.422***	0.425***	0.424	0.458	0.286***	0.661
	80+		1.105***	1.108***	0.883**	1.688***	0.916***	1.602**
Handgrip strength	High		−0.365***	−0.365***	−0.770	0.525		
	Missing		0.0744**	0.0765**	0.165	0.161		
	Continous						−0.0206***	−0.0426**
ln p		0.173***	0.216***	0.218***	0.194	0.0787	0 222***	0.140
Sample size		33379	33379	33174	106	99	31078	90

Note: Left out categories are no cancer, male, 50–59 years, and low HGS.

* p<0.05,

** p<0.01,

*** p<0.001

## Data Availability

The data is publicly available after application from https://share-eric.eu/. Access to data requires an individual’s free registration, followed by the acceptance of the SHARE Conditions and signing the SHARE User Statement. After acceptance of these documents, data can be downloaded using the personal ID and password.
